# Identification of Potential Molecular Mechanisms and Candidate Genes Involved in The Acute Phase of Myocardial Infarction

**DOI:** 10.22074/cellj.2018.5213

**Published:** 2018-05-28

**Authors:** Yushuang Yang, Jie Yang, Fenghua Sui, Pengfei Huo, Hailing Yang

**Affiliations:** 1 Department of Cardiovascular, China-Japan Union Hospital, Jilin University, Changchun, China; 2 Department of Endocrinology, China-Japan Union Hospital, Jilin University, Changchun, China; 3 Cardiovascular Medicine, China-Japan Union Hospital (Xinmin District), Jilin University, Changchun, China; 4 Intensive Care Unit, China-Japan Union Hospital, Jilin University, Changchun, China; 5 Department of Emergency, China-Japan Union Hospital, Jilin University, Changchun, China

**Keywords:** Gene Expression Profile, Myocardial Infarction, Protein-Protein Interaction Network, Transcriptional Regulatory Network

## Abstract

**Objective:**

This study used bioinformatics to determine genetic factors involved in progression of acute myocardial infarction (MI).

**Materials and Methods:**

In this prospective study, gene expression profile GSE59867 was downloaded from the Gene Expression Omnibus database, which contained 46 normal samples obtained from stable coronary artery disease patients (n=46) who were without history of MI (control) and 390 samples from patients (n=111) who had evolving ST-segment elevation myocardial infarction (STEMI) as the MI group. These samples were divided into 4 groups based on time points. After identification of differentially expressed genes (DEGs), we conducted hierarchical clustering and functional enrichment analysis. Protein interaction and transcriptional regulation among DEGs were analysed.

**Results:**

We observed 8 clusters of DEGs that had a peak or a minimum at the t=1 time point according to gene expression levels. Upregulated DEGs showed significant enrichment in the biological process, single-organism cellular process, response to stimulus and stress, and osteoclast differentiation and lysosome. Downregulated DEGs enriched in the T-cell receptor signalling pathway and natural killer cell mediated cytotoxicity. We identified multiple genes, including signal transducer and activator of transcription 3 (STAT3); LCK proto-oncogene, Src family tyrosine kinase (LCK); and FYN proto-oncogene, Src family tyrosine kinase (FYN) from the protein-protein interaction (PPI) network and/or the transcriptional regulatory network.

**Conclusion:**

Cytokine-mediated inflammation, lysosome and osteoclast differentiation, and metabolism processes, as well as STAT3 may be involved in the acute phase of MI.

## Introduction

Myocardial infarction (MI) is usually caused by clot formation in the coronary arteries which stops the blood flow, then leads to heart muscle cell death as a consequence of oxygen deprivation (1). It is one of the leading causes of disability and death in the world. An estimated one million people experience an MI each year in the United States (2). Although advanced therapies following MI could improve short-term survival, the incidence of heart failure, recurrence, and long-term mortality is steadily increasing worldwide (3).

Some genetic variants associated with the increased risk of MI were discovered via genome-wide analysis. For example, a common *ABO* genetic variation linked to the ABO blood group system might modulate various distinct pathways and protect against MI (4). Atherosclerosis has a gradual onset due to the buildup of atherosclerotic plaques over a long period of time, whereas symptoms of MI are acute (5). Complications of MI such as heart failure and atrial fibrillation may take time to develop. Atherosclerotic plaques can significantly increase the accumulation and recruitment of leukocytes, which are common results of an MI and increase the risk of re-infarction (6). In addition, emergency haematopoiesis and local environmental changes in the spleen can occur (7). However, the molecular mechanisms before and after MI are largely unknown. 

To determine genetic factors involved in progression of acute MI, we applied microarray data collected from MI patients at several time points to identify candidate genes and their potential roles in the risk stratification following MI. This study might provide insight into the treatment optimized to improve the outcome of an acute MI. 

## Materials and Methods

### Microarray data and samples

In this prospective study, the expression profiling GSE59867 generated by Maciejak et al. (8) from peripheral blood mononuclear cells were obtained from the Gene Expression Omnibus (GEO) database. This microarray data consisted of 46 normal samples from stable coronary artery disease patients (n=46) who did not have a history of MI (control group) and 390 MI samples from patients (n=111) with evolving ST-segment elevation MI (STEMI). These MI samples were divided into four groups based on time points: 1st day after MI (t=1, admission), 4-6 days after MI (t=2, discharge), 1 month after MI (t=3), and 6 months after MI (t=4). This data was sequenced on the platform of GPL6244 [Affymetrix Human Gene 1.0 ST Array, transcript (gene) version; Affymetrix, Santa Clara, CA, USA]. The construction of this dataset was authorized by the local Ethics Committees of the Medical University of Warsaw and Medical University of Bialystok, and guided by the principles of the Declaration of Helsinki. All participants provided informed consents were obtained from all participants.

### Data pre-processing and annotation 

The expression matrix retrieved from the GEO database was pre-processed by the robust multiarray analysis (RMA) method and expression values were log2 transformed. Genes represented by probe sets were annotated, and the average signal level of probes were defined as the expression levels of the genes. 

### Identification of differentially expressed genes and hierarchical clustering analysis

We performed one-way analysis of variance for differential expression between the 4 time series datasets compared to the controls (t=0) to identify the differentially expressed genes (DEGs). P values were adjusted by the Benjamini Hochberg (BH) method. Genes with P<1×10^-6^ were considered to be differentially expressed. 

Noise robust soft clustering analysis of time series gene expression data were conducted by The R package Mfuzz (9) (http://itb1.biologie.hu-berlin. de/~futschik/ software/R/Mfuzz/index.html) using a fuzzy c-means algorithm. Then, we divided genes involved in multiple clusters into two sections according to expression (high and low). Parameters used in this study were cluster membership (MEM. SHIP)=0.5 and cluster number=8.

### Functional enrichment analysis 

R package clusterProfiler (10) is commonly used to analyse and visualize functional classifications for genes and gene clusters. It has been used to determine the Kyoto Encyclopedia of Genes and Genomes (KEGG) pathways and the Gene Ontology-Biological Process (GO-BP) enriched by DEGs. These enrichment significances were adjusted by BH procedure and statistical significant was considered with an adjusted P<0.05.

### Analysis of protein-protein interactions

Protein interactions were obtained using the Search Tool for the Retrieval of Interacting Genes (STRING) (11) database source, which provides experimental and predicted protein interaction information. The computed combined score (0-1) indicates higher confidence when the value is high. The criteria of the combined score was set to no less than 0.9 to keep the network analysis of protein-protein interaction (PPI) manageable. The PPI network was visualized in Cytoscape (12).

### Analysis of transcriptional regulation 

The Encyclopedia of DNA Elements (ENCODE) project is an international research consortium that uses large-scale, genome-wide assays to identify the role of all functional elements of the human genome (13). The transcription regulatory association between DEGs were explored by ENCODE, and the network was displayed by the Cytoscape (12) plug.

## Results

### Differentially expressed genes and clusters 

**Fig.1:**
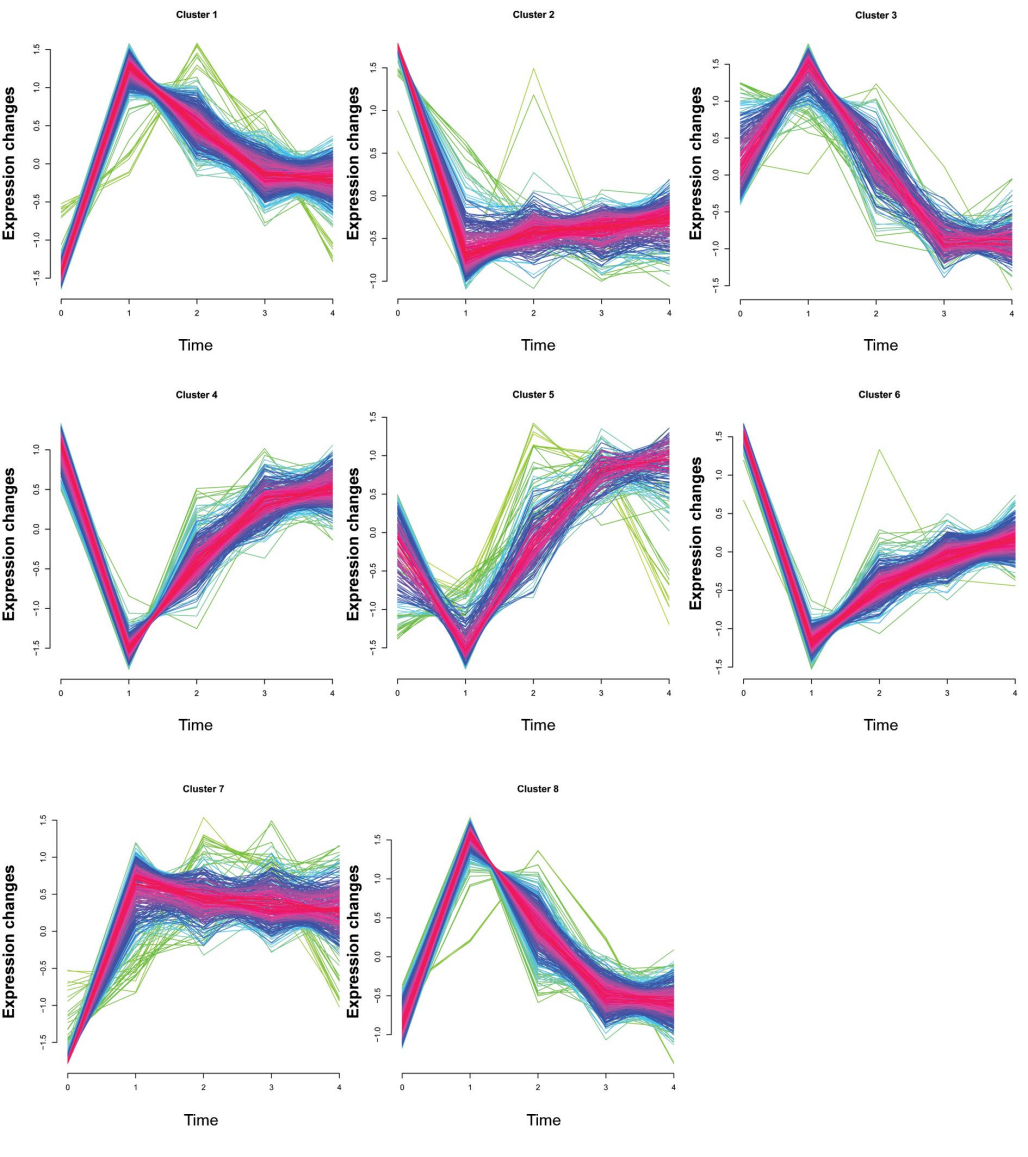
Gene clustering results (time-series plots) based on gene expression levels. The horizontal axis denotes time points [0 day, day 1, 4-6 days, 1 month, and 6 months after myocardial infarction (MI)]. The vertical axis denotes the expression level (log 2 (ratio). Red indicates the variation of gene is more conformed to the center of the cluster, followed by blue, and finally green.

Eight clusters of DEGs were shown in Figure 1. Each gene in 8 clusters had an acute peak or a valley expression that occurred at the t=1 time point, whereas during t=2 to t=4, their expressions tended to stabilize or approached the expression level at t=0. The genes with valley expressions were supposed to be down regulated following MI, which included cluster 2, cluster 4, cluster 5, and cluster 6 with genes 264, 289, 123, and 461, respectively. Those with peaks were highly-expressed (up) following the MI (Fig . 1).

### Functional terms enriched by differentially expressed genes 

The significant enriched GO-BP terms and pathways enriched by up-and down-regulated DEGs were shown in Table 1. Upregulated DEGs were mainly associated with biological processes, single-organism cellular processes, response to stimulus and stress, and enriched in pathways of osteoclast differentiation, lysosome, leishmaniasis, glycolysis and several signalling pathways. In addition to biological and cellular processes, GO-BP terms of multiple metabolic processes were related to functions of downregulated DEGs. The T-cell receptor signalling pathway, natural killer cell mediated cytotoxicity, ubiquitin mediated proteolysis, and B-cell receptor signalling pathway were enriched by downregulated DEGs. 

### Protein-protein interaction network of differentially expressed genes 

**Fig.2:**
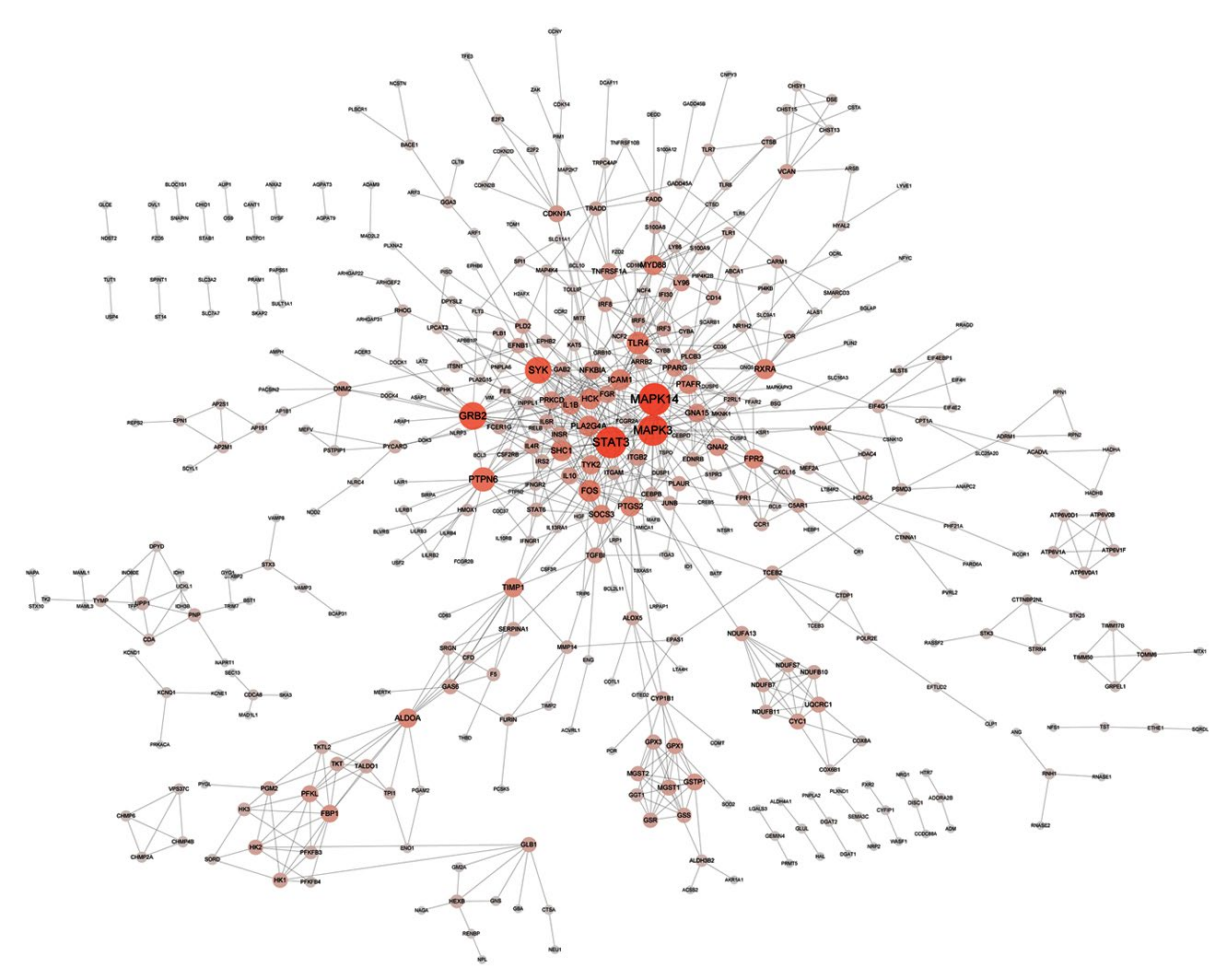
Protein-protein interaction (PPI) network of upregulated differentially expressed genes (DEGs). The brighter red color and larger size of a node represent a higher degree of the node. Edges represent the connection between nodes.

**Fig.3:**
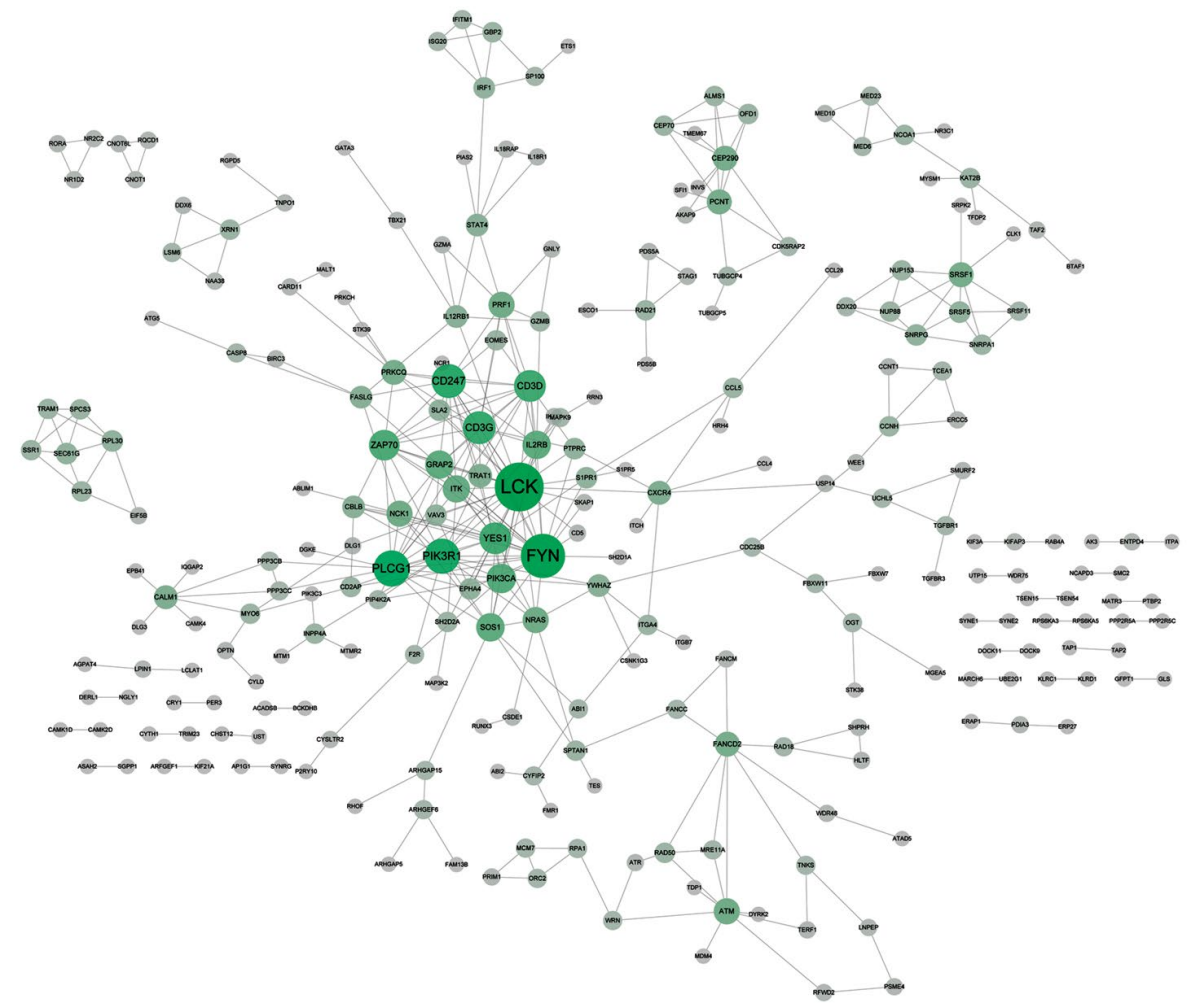
Protein-protein interaction (PPI) network of downregulated differentially expressed genes (DEGs). The brighter green color and larger size of a node represent a higher degree of the node. Edges represent the connection between nodes.

A PPI network constructed from 788 protein pairs across 440 upregulated DEGs were shown in Figure 2. The r-squared score was 0.914. The PPI network displayed in Figure 3 was composed by 256 downregulated DEGs and 401 interactions between them, with an r-squared score of 0.902. Therefore, these two networks were well approximate to scale-free network (SFN), which had few node with higher degree than nearby one and played critical regulatory role network. The top 5 hub nodes that included upregulated genes of mitogen-activated protein/ERK kinase kinases (*MAPK14*); signal transducer and activator of transcription 3 (*STAT3*); and mitogen-activated protein kinases (*MAPK3*); in addition downregulated genes of LCK proto-oncogene, Src family tyrosine kinase (*LCK*); FYN proto-oncogene, Src family tyrosine kinase (*FYN*); and phospholipase C, gamma 1 (*PLCG1*) were identified based on the degree in the network (Table 2). 

**Table 1:** Gene ontology (GO) and the Kyoto encyclopedia of genes and genomes (KEGG) pathway enrichment analyses of differentially expressed genes (DEGs)


ID	Description	p.adjust

Upregulated genes		
GO:0008150	Biological_process	4.82E-90
GO:0044699	Single-organism process	4.52E-51
GO:0009987	Cellular process	8.91E-43
GO:0044763	Single-organism cellular process	1.63E-41
GO:0050896	Response to stimulus	1.65E-32
GO:0002376	Immune system process	1.09E-27
GO:0006950	Response to stress	4.51E-25
hsa04380	Osteoclast differentiation	4.98E-10
hsa04142	Lysosome	4.44E-09
hsa05140	Leishmaniasis	8.19E-06
hsa00010	Glycolysis/gluconeogenesis	8.19E-05
hsa04145	Phagosome	5.22E-04
Downregulated genes		
GO:0008150	Biological process	1.76E-71
GO:0009987	Cellular process	4.02E-45
GO:0044260	Cellular macromolecule metabolic process	7.63E-35
GO:0044237	Cellular metabolic process	9.19E-32
GO:0044238	Primary metabolic process	9.22E-32
GO:0071704	Organic substance metabolic process	1.44E-30
GO:0043170	Macromolecule metabolic process	5.91E-30
hsa04660	T-cell receptor signaling pathway	2.58E-09
hsa04650	Natural killer cell mediated cytotoxicity	1.89E-05
hsa04120	Ubiquitin mediated proteolysis	8.47E-03
hsa04662	B-cell receptor signaling pathway	1.30E-02
hsa05340	Primary immunodeficiency	1.30E-02


**Table 2:** Top 5 hub nodes in the up- and downregulated protein-protein interaction (PPI) network


Gene	Up/down	Degree

*MAPK14*	Up	28
*STAT3*	Up	27
*MAPK3*	Up	26
*GRB2*	Up	22
*SYK*	Up	21
*LCK*	Down	29
*FYN*	Down	25
*PLCG1*	Down	18
*PIK3R1*	Down	17
*CD247*	Down	16


### Transcriptional regulatory network of differentially expressed genes 

**Fig.4:**
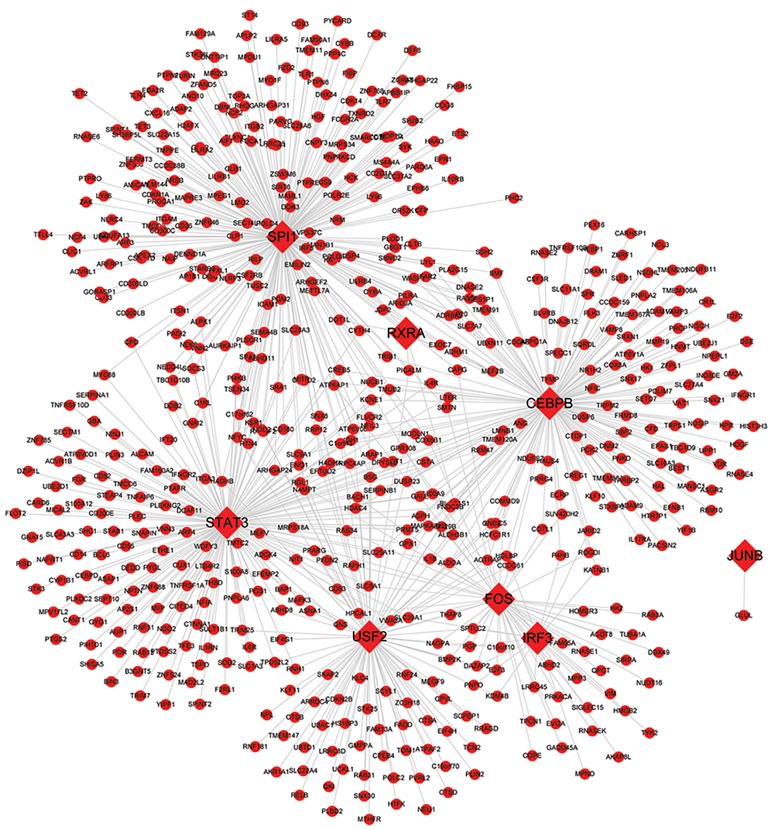
Transcriptional regulatory network of upregulated differentially expressed genes (DEGs). Rhombus nodes denote transcription factors (TFs), and circle nodes represent non-TF genes.

**Fig.5:**
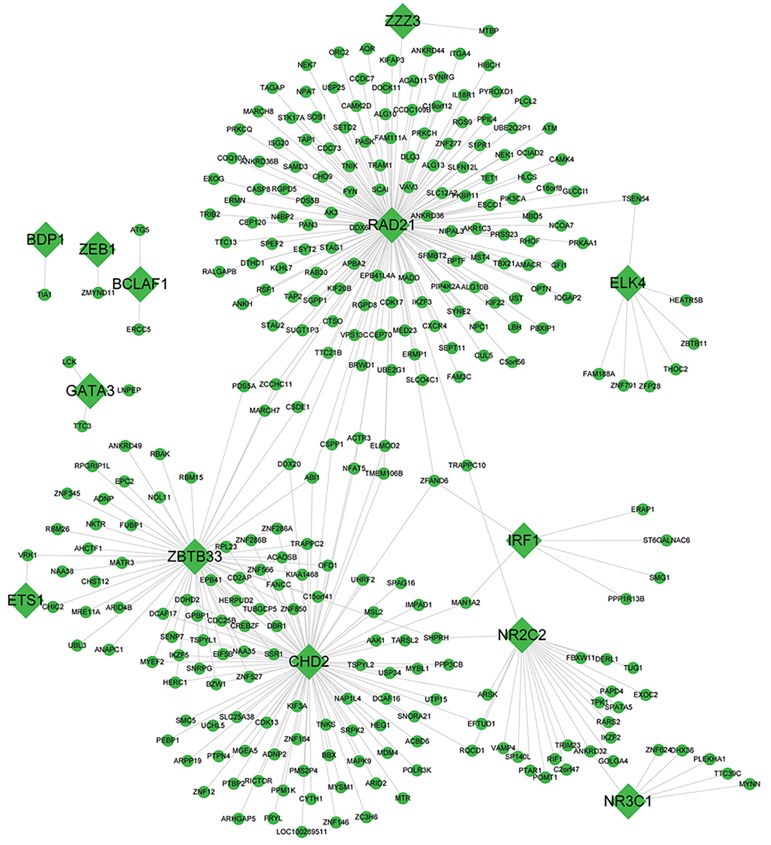
Transcriptional regulatory network of downregulated differentially expressed genes (DEGs). Rhombus nodes denote transcription factors (TFs), and circle nodes represent non-TF genes.

The transcriptional regulatory network of up- and down- regulated DEGs were shown in Figure 4 and Figure 5, respectively. Transcription factors (TFs) exert dominant roles in the transcriptional regulatory network. The central TFs and degree of them were summarized in Table 3. In particular, the upregulated DEGs of spi-1 proto-oncogene (*SPI1); STAT3;* and CCAAT/enhancer-binding proteins (*CEBPB*) had high degrees of connectivity. 

**Table 3:** Transcription factor (TFs) gene with high degree in the transcriptional regulatory network


TFs	Up/down	Degree

SPI1	Up	240
STAT3	Up	185
CEBPB	Up	165
RAD21	Down	146
USF2	Up	103
CHD2	Down	90
ZBTB33	Down	58
FOS	Up	52
NR2C2	Down	23
IRF3	Up	15
RXRA	Up	7
ELK4	Down	7
NR3C1	Down	6
IRF1	Down	6


Up; Upregulated and Down; Downregulated.

## Discussion

This gene expression profile was explored by Maciejak et al. (8) to assess potential prognostic biomarkers for heart failure development following MI. In our study, different analysis criteria and methods that included hierarchical clustering analysis were performed on the original data. TFs which generally play essential roles in the regulation of critical biological processes and affect the host regulatory networks in different cell types have been explored from the DEGs list. 

Our study might provide some other theoretical perspective for better prognosis of MI. 

DEGs obtained in the current study were clustered into 8 clusters. We observed significant changes in gene expression in all clusters at the time point of the first day of the MI. A previous study suggested that the serum level of multiple plasma components that included brain natriuretic peptide acutely increased in the early phase (2fold at 12 hours) and increased until 3 days after the AMI (14, 15). Previous studies showed that cytokine-mediated inflammation was activated and played an important role in the early stage of an MI (16). In addition, the aberrantly expressed miRNA signatures, some of which might have defensive effects in target gene mediated cardiac protection, could illustrate the huge pathophysiologic response to AMI in the early phase of an AMI (17). 

According to the functional enrichment analysis, biological process categories that include response to stimulus and stress were activated, which was consistent with the studies described above. In addition, we identified upregulated pathways of osteoclast differentiation and lysosomes. The lysosome was disrupted and we observed increased plasma lysosome enzyme activity before the inflammatory reaction (18). 

Lysosomal proteases that produce bioactive fragments were proven to have a role in post-MI remodelling (19). However, there were few studies about the correlation between the prior pathway and MI. MI has been associated with variations within the interleukin-23 receptor (IL23R) which participates in chronic inflammation and inhibits osteoclastogenesis (20). In addition, the level of serum osteoprotegerin was found to be increased in coronary artery disease and future cardiovascular events (21). Therefore, in addition to lysosome and cytokine-mediated inflammation, osteoclast differentiation might have a close interaction with MI. 

Some genes, including *STAT3*, were identified through the construction of the PPI network and the transcriptional network. IL-10-mediated suppressed inflammatory response and increased angiogenesis after MI, which might be through the activation of *STAT3* (22). *STAT3* deletion in subacute MI exacerbated cardiac hypertrophy and cardiac remodelling (23). Therefore, *STAT3* could be activated to deliver a survival signal to the ischemic preconditioning of myocardium. 

Moreover, GO categories of cellular metabolic processes and pathways that include the T-cell receptor signalling pathway, natural killer cell mediated cytotoxicity, ubiquitin mediated proteolysis, and the B-cell receptor signalling pathway were enriched by downregulated DEGs. These findings supported the results of Xie et al. (24). Studies have found that urine microRNAs, which delineate important perturbations in metabolic processes, such as miR-1 abnormally expressed in acute MI (25). 

*LCK* and *FYN* encoded proteins are members of the Src family tyrosine kinases. The former plays a key role in the selection as well as maturation of developing T-cells (26), while the latter is involved in downstream signalling pathways that result in T-cell differentiation and proliferation (27). In addition, *RAD21*, as a TF encoding gene, is the hub node in the transcriptional regulatory network. It is a critical protein in chromosome cohesion during the cell cycle and cell apoptosis. Cell cycle activation could partially counteract with cardiac function and the adverse ventricular remodelling after MI (28). However, in the acute phase, it is reversed. Apoptosis has been reported at the acute stage of MI to determine the final infarct size (29). Therefore, molecular metabolism processes, the cell cycle, and cell apoptosis could also participant in the acute stage of MI. Furthermore, LCK and *FYN* as well as the TF gene, *RAD21*, could play important roles in MI. 

## Conclusion

Upregulated pathways that include lysosome and cytokine-mediated inflammation, osteoclast differentiation, and STAT3 might have close interactions with an acute MI. Besides molecular metabolism processes, the cell cycle and cell apoptosis were disturbed. Genes such as LCK, FYN, and RAD21 could also participant in the acute stage of MI. However, there were some limitations in our current study, and more experiments would be necessary to validate these results. 
